# Author Correction: Ferroelectric ultraviolet photodetector material with ultrafast response speed

**DOI:** 10.1038/s41467-026-78436-6

**Published:** 2026-10-07

**Authors:** Xuexi Yan, Tingting Yan, Lingli Li, Yi Cao, Xinwei Wang, Jinghui Wang, Ang Tao, Tingting Yao, Yixiao Jiang, Weijin Hu, Xiaosheng Fang, Hengqiang Ye, Xiu-Liang Ma, Chunlin Chen

**Affiliations:** 1 Shenyang National Laboratory for Materials Science, Institute of Metal Research, Chinese Academy of Sciences, School of Material Science and Engineering, University of Science and Technology of China, 110016 Shenyang, China; 2 College of Smart Materials and Future Energy, State Key Laboratory of Molecular Engineering of Polymers, Fudan University, 200433 Shanghai, China; 3 Ji Hua Laboratory, 528200 Foshan, China; 4 Bay Area Center for Electron Microscopy, Songshan Lake Materials Laboratory, 523808 Dongguan, China; 5 Institute of Physics, Chinese Academy of Sciences, 100190 Beijing, China; 6 State Key Lab of Advanced Processing and Recycling on Non-ferrous Metals, Lanzhou University of Technology, 730050 Lanzhou, China

Correction to: *Nature Communications* https://doi.org/10.1038/s41467-025-68069-6, published online 30 December 2025

In the version of the article initially published, in the seventh paragraph of the Results and discussion, and in the legend for Supplementary Fig. 21, the text “Their rise/fall times are 6.6 ns/17.7 ns and 8.7 ns/12.2 ns, respectively” should have read “Their rise/fall times are 6.6 ns/14.7 ns and 8.7 ns/12.2 ns, respectively”. This correction has been made to the HTML and PDF versions of the article.

