# Supplementary material for: Ferroelectric ultraviolet photodetector material with ultrafast response speed
Source: Nat Commun. 2025 Dec 30;17:1304. doi: 10.1038/s41467-025-68069-6 (PMC12868875; doi:10.1038/s41467-025-68069-6)
Supplement: Supplementary file 1 — Supplementary Information [file 41467_2025_68069_MOESM1_ESM.pdf]

Supplementary Information for

Ferroelectric ultraviolet photodetector material with ultrafast response speed

Xuexi Yan[1,▽], Tingting Yan[2,▽], Lingli Li[1], Yi Cao[1], Xinwei Wang[1], Jinghui Wang[1], Ang Tao[1], Tingting Yao[1], Yixiao Jiang[1], Weijin Hu[1], Xiaosheng Fang[2], Hengqiang Ye[3], Xiu-Liang Ma[4,5,6,*] & Chunlin Chen[1,*]

[1] Shenyang National Laboratory for Materials Science, Institute of Metal Research, Chinese Academy of Sciences, School of Material Science and Engineering, University of Science and Technology of China, Shenyang 110016, China

[2] Department of Materials Science, Fudan University, Shanghai 200433, China

[3] Ji Hua Laboratory, Foshan 528200, China

[4] Bay Area Center for Electron Microscopy, Songshan Lake Materials Laboratory, Dongguan 523808, China

[5] Institute of Physics, Chinese Academy of Sciences, Beijing 100190, China

[6] State Key Lab of Advanced Processing and Recycling on Non-ferrous Metals, Lanzhou University of Technology, 730050 Lanzhou, China

[*] Corresponding author: Chunlin Chen (clchen@imr.ac.cn), Xiu-Liang Ma (xlma@iphy.ac.cn)

[▽] Xuexi Yan and Tingting Yan contributed equally to this work.

Supplementary Figure S1

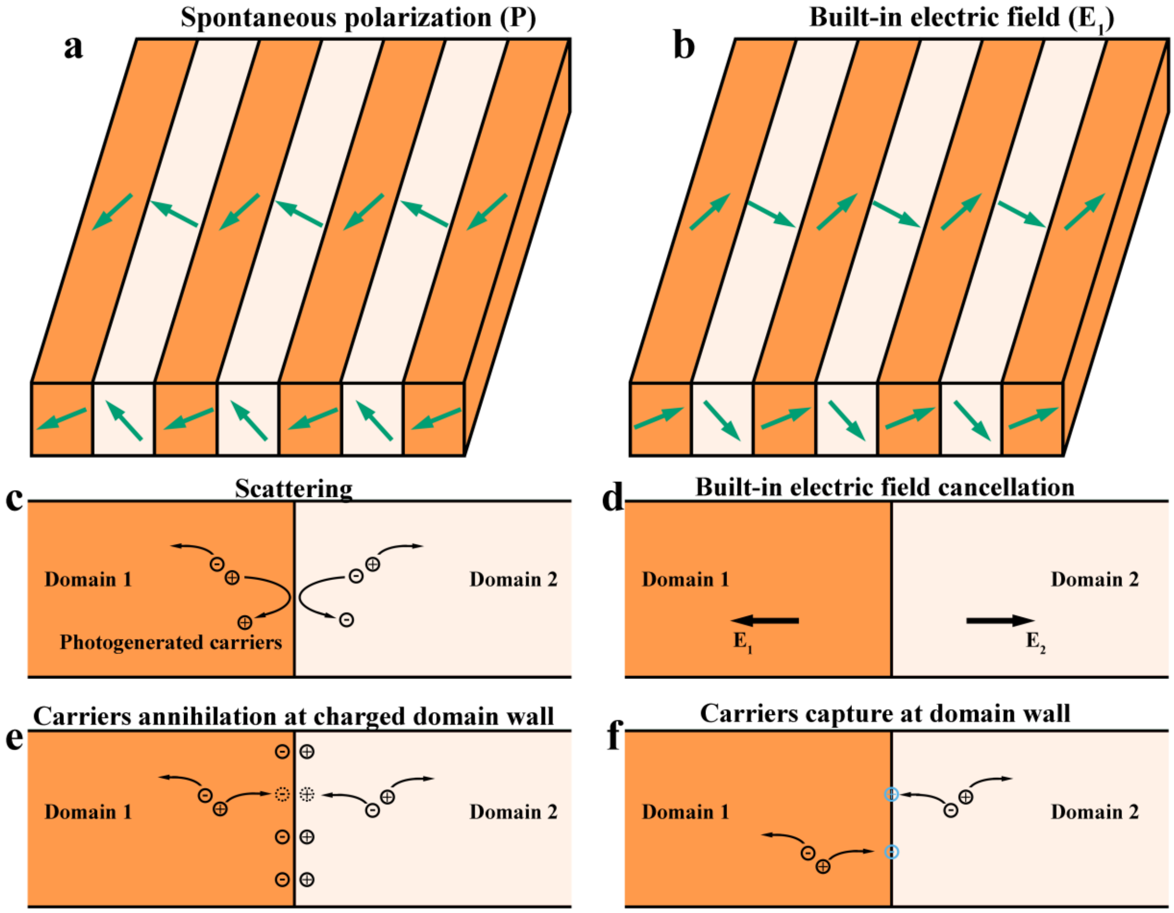

Supplementary Fig. S1 | Schematic diagram of the performance failure of traditional ferroelectric photodetectors. (a) Spontaneous polarization and **(b)** built-in electric field distribution in traditional ferroelectric materials. Most of ferroelectric materials have a high density of ferroelectric domains with different polarization directions. **(c-f)** Several common failure mechanisms of ferroelectric photodetectors include scattering, annihilation, capture of photogenerated carriers at domain walls, and cancellation of built-in electric fields between different domains.

Supplementary Figure S2

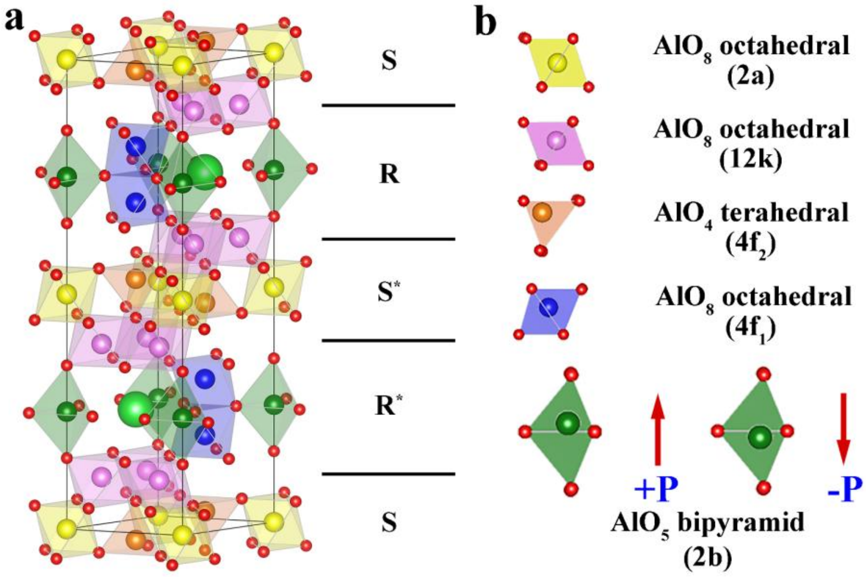

Supplementary Fig. S2 | Schematic diagram of the atomic model of SATO. (a) Atomic model of SATO unit cell, which comprises alternating S and R blocks in the sequence of SRS^*R^* (* denotes rotating that layer around the c axis by 180°). SATO is a common magnetoplumbite structure with a space group of $P6_3/mmc$. **(b)** The corresponding Wyckoff positions occupied by Al and Ti atoms. Al atoms occupy the 2a, 12k, $4f_2$, $4f_1$ and 2b symmetric positions, and Ti atoms partially replace the Al atoms at the $4f_1$ position. The misalignment of the positive and negative charge centers in the AlO_5 bipyramid makes the SATO exhibit ferroelectricity.

Supplementary Figure S3

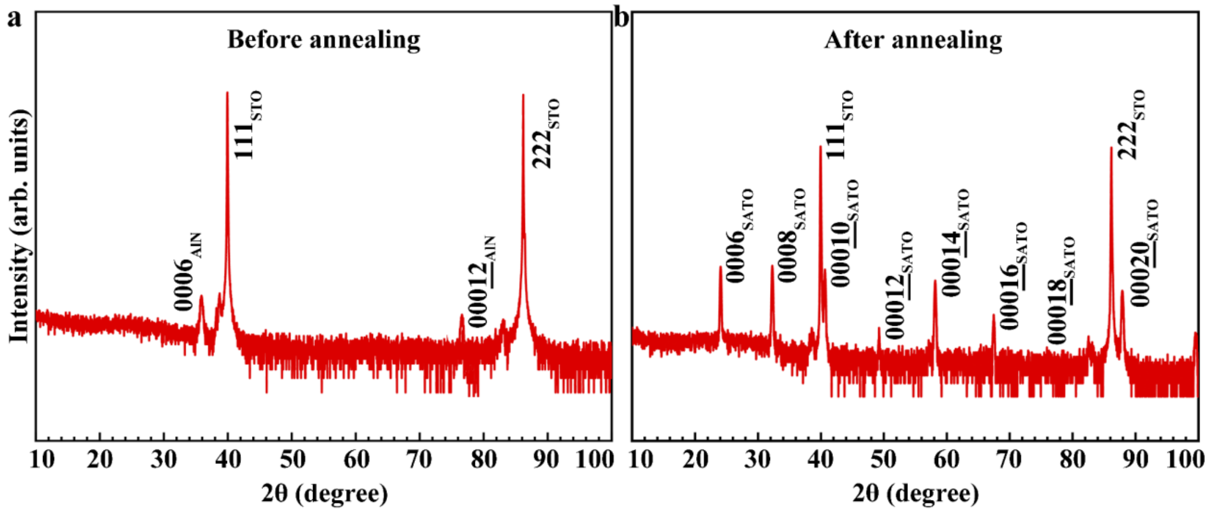

Supplementary Fig. S3 | (a) Out-of-plane HRXRD pattern of as-prepared AlN thin film on the STO (111) substrate. The AlN film was epitaxially grown on the STO substrate with orientation relationship of AlN (0006) // STO (111). **(b)** Out-of-plane HRXRD pattern of the thin film after annealing at 1500°C in air. The SATO thin film was formed due to the solid-state reaction of AlN and STO. The epitaxial relationship is STAO (0006) // STO (111).

Supplementary Figure S4

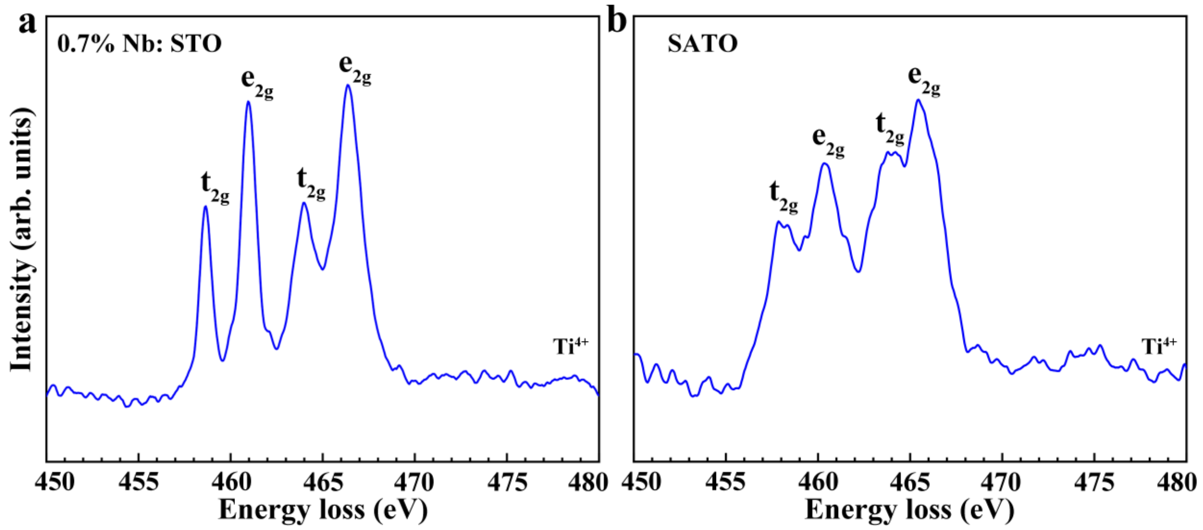

Supplementary Fig. S4 | EELS spectra showing the Ti $L_{2,3}$ edges in STO and SATO. (a, b) The Ti $L_{2,3}$ edges of SATO and STO have very similar fine structures with four peaks, suggesting that the Ti ions in SATO have the valence state of +4. (a, b)

Supplementary Figure S5

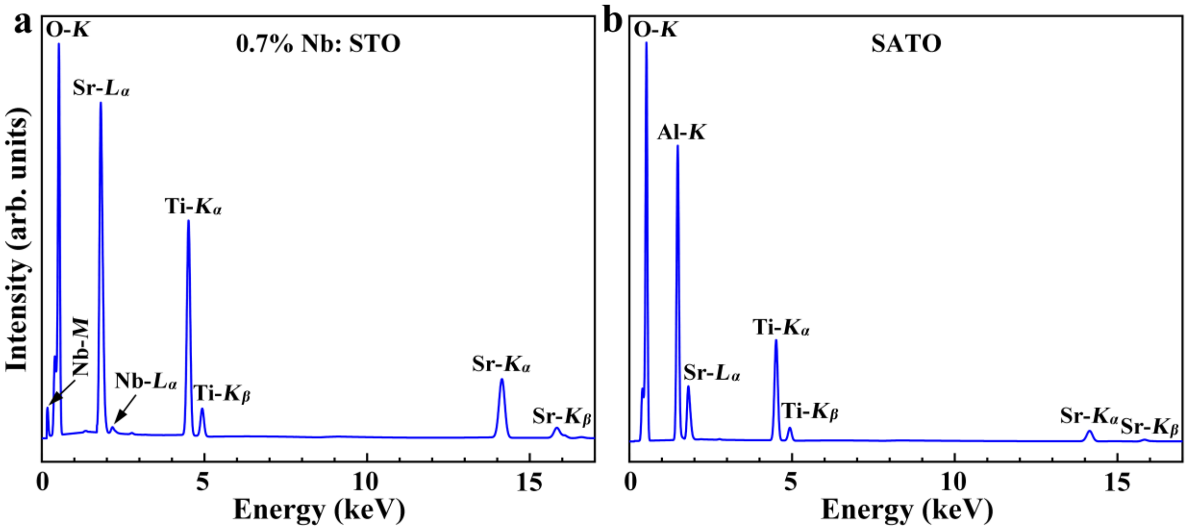

Supplementary Fig. S5 | EDS spectra recorded from the Nb-doped (0.7at.%) STO substrate and the SATO thin film. (a, b) Only Sr, Al, Ti, and O elements are detected in the SATO thin film. Nb did not diffuse from the substrate to the SATO thin film.

Supplementary Figure S6

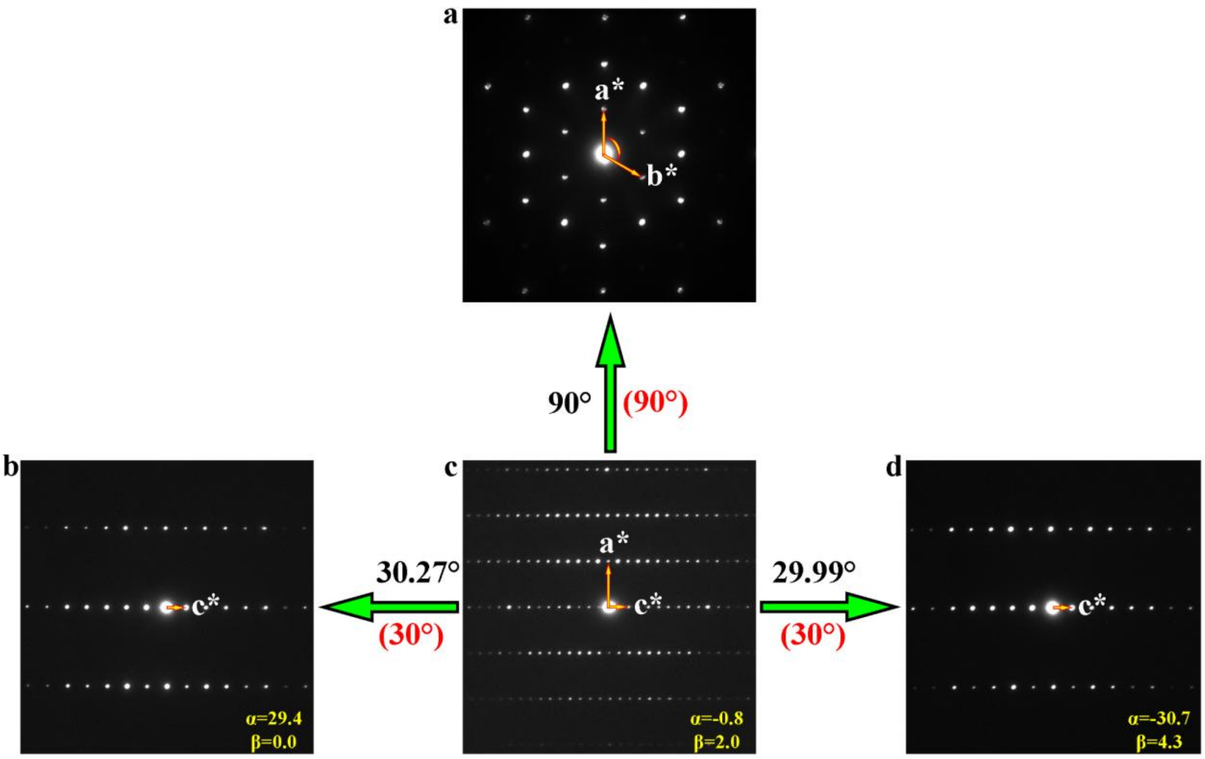

Supplementary Fig. S6 | Tilt-series of SAED patterns of the STAO thin film. (a-d) The diffraction characteristics and extinction law are consistent with the structure of magnetoplumbite. α and β represent the tilt angles of the TEM goniometer. The theoretical values of the tilt angle between the adjacent zone axes are highlighted by red font in the brackets, and the experimentally measured value were denoted by black font.

Supplementary Figure S7

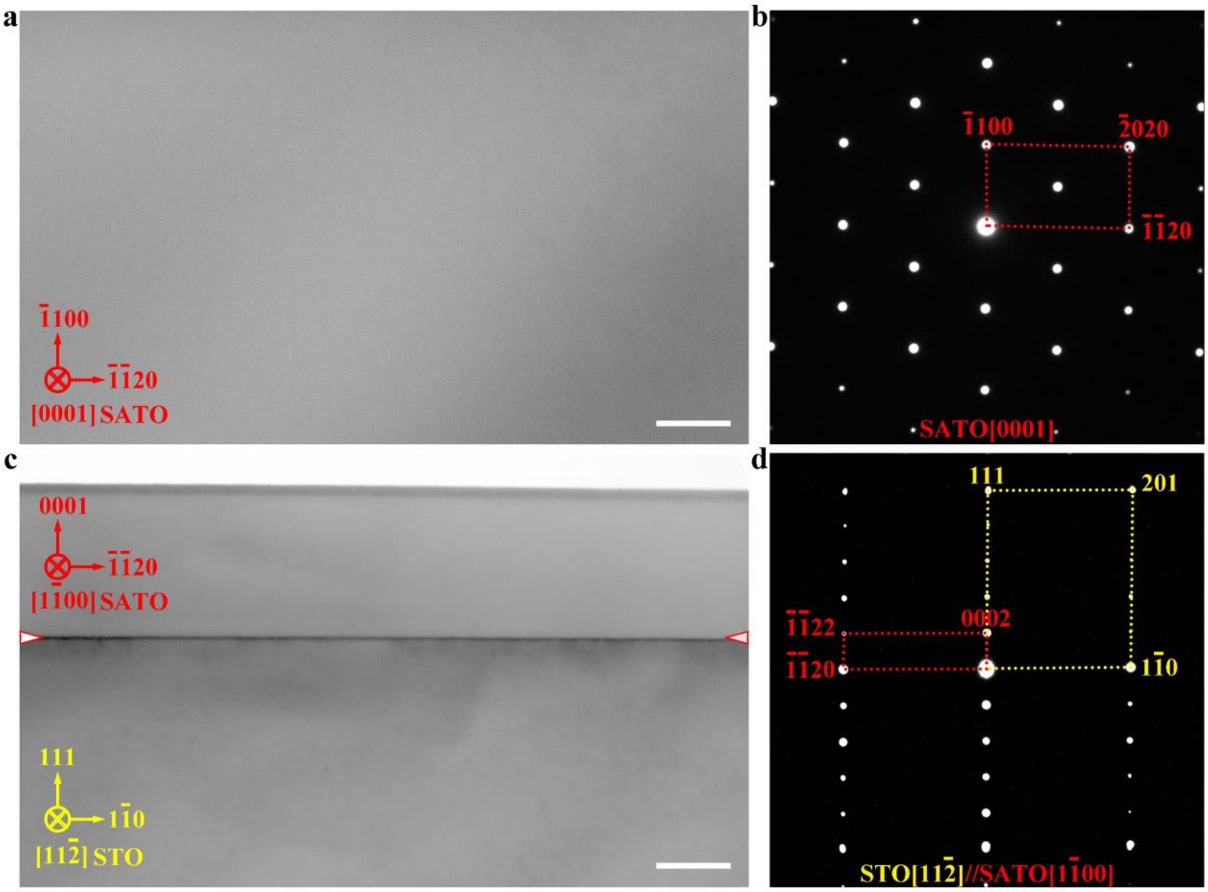

Supplementary Fig. S7 | Microstructure of the SATO thin film observed from two orthogonal directions. (a) Bright-field TEM image and **(b)** corresponding SAED pattern of the SATO thin film along the SATO [0001] direction. **(c)** Bright-field TEM image and **(d)** corresponding SAED pattern of the STAO thin film along the SATO $[1\bar{1}00]$ direction. The uniform contrast of the film indicates that the SATO ferroelectric film exhibits the possibility of single-domain ferroelastics. No ferroelectric domain walls can be observed. Scale bar, 20 nm.

Supplementary Figure S8

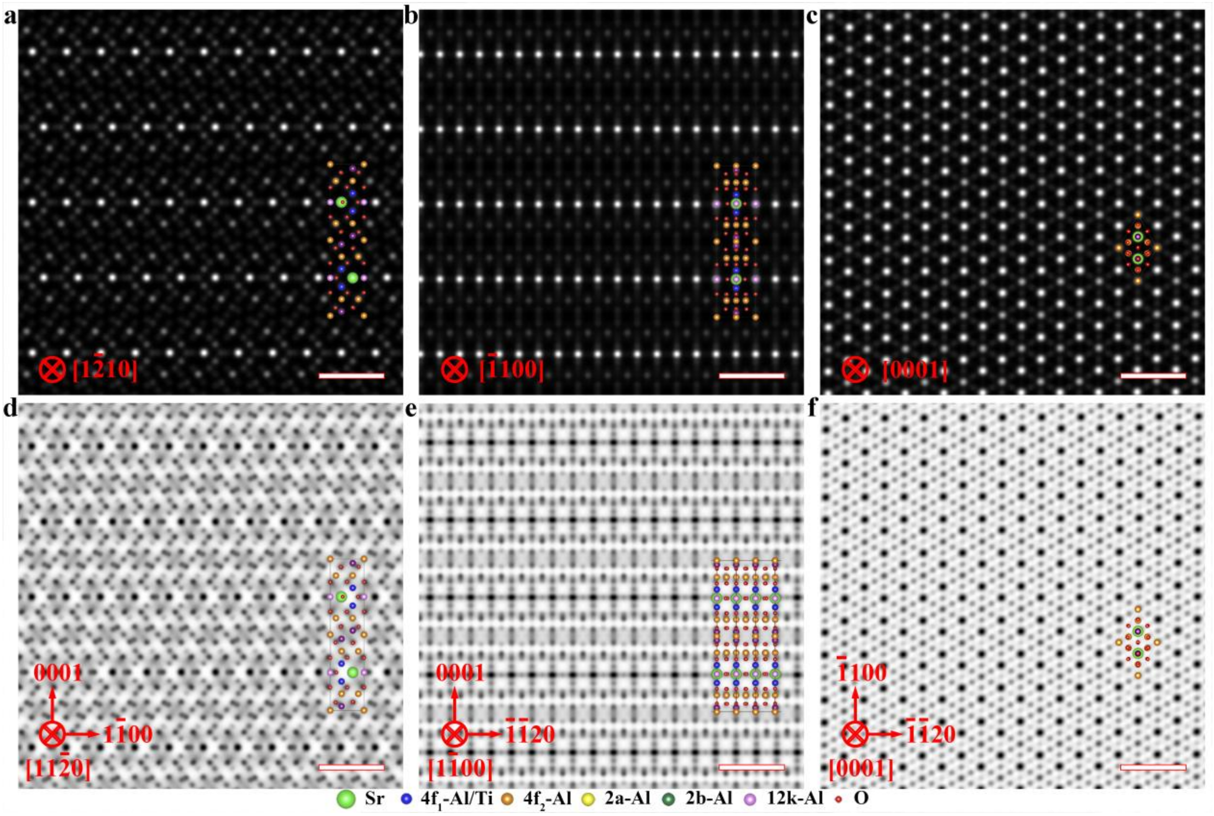

Supplementary Fig. S8 | Simulated HAADF and ABF STEM images. (a-c) Simulated HAADF STEM images of STAO along the [11$\bar{2}$0], [1$\bar{1}$00], and [0001] zone axes. **(d-f)** Corresponding simulated ABF STEM images. Scale bar, 1 nm.

Supplementary Figure S9

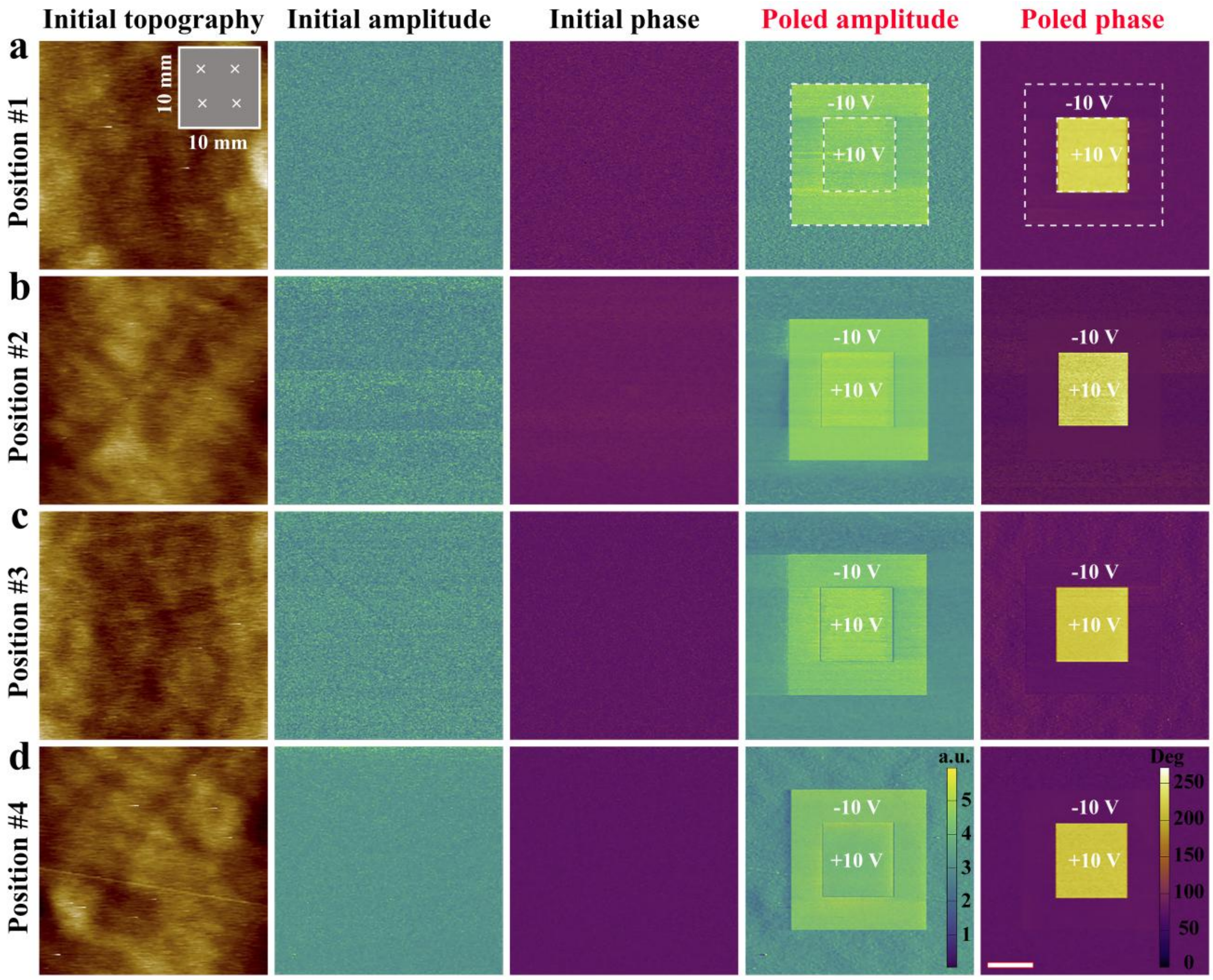

Supplementary Fig. S9 | PFM characterizations of the SATO thin films. (a-d) PFM topography, amplitude, and phase images over 10 × 10 μm^2 at four different positions. The inset in **a** shows the measurement positions. Both the initial amplitude and phase images of the SATO film exhibit a uniform contrast under 0 V and -10 V, indicating that the SATO film exhibits the possibility of single-domain ferroelectrics with the polarization along out-of-plane direction. Scale bar, 2 μm.

Supplementary Figure S10

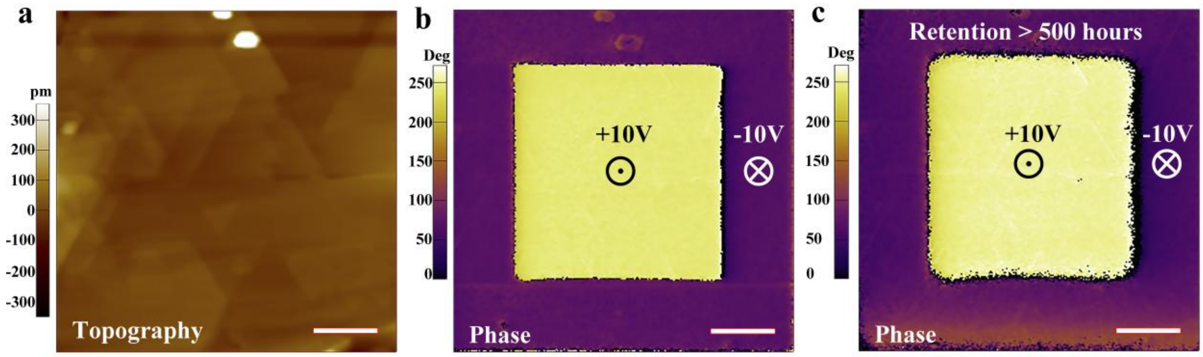

Supplementary Fig. S10 | PFM retention measurements of the SATO thin film. (a) PFM topographic image of the SATO thin film. **(b,c)** The out-of-plane phase images of electrical poled domains. The written domains can be maintained for > 500 h without noticeable changes, demonstrating robust ferroelectric stability. Scale bar, 2 μm.

Supplementary Figure S11

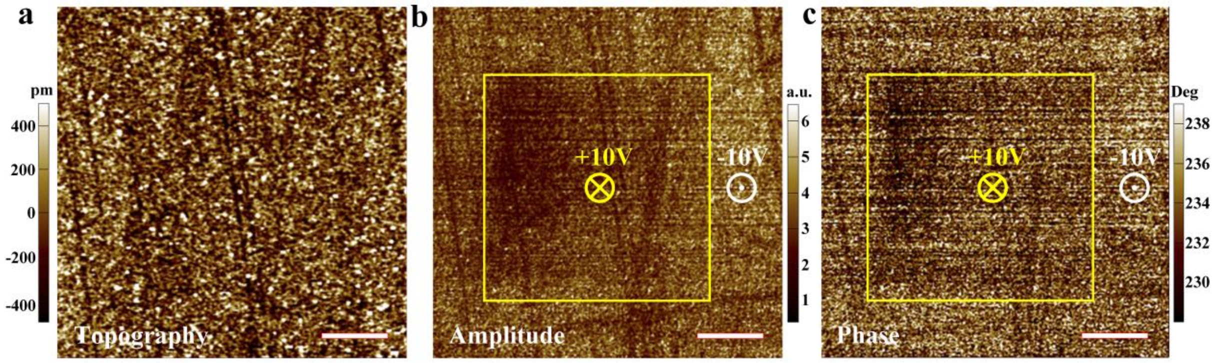

Supplementary Fig. S11 | PFM characterizations of the AlN thin film on the Nb:STO (111) substrate. (a-c) PFM topography, amplitude, and phase images of the AlN film. Domains cannot be written in the AlN film, suggesting that the AlN film is not ferroelectric. Scale bar, 2 μm.

Supplementary Figure S12

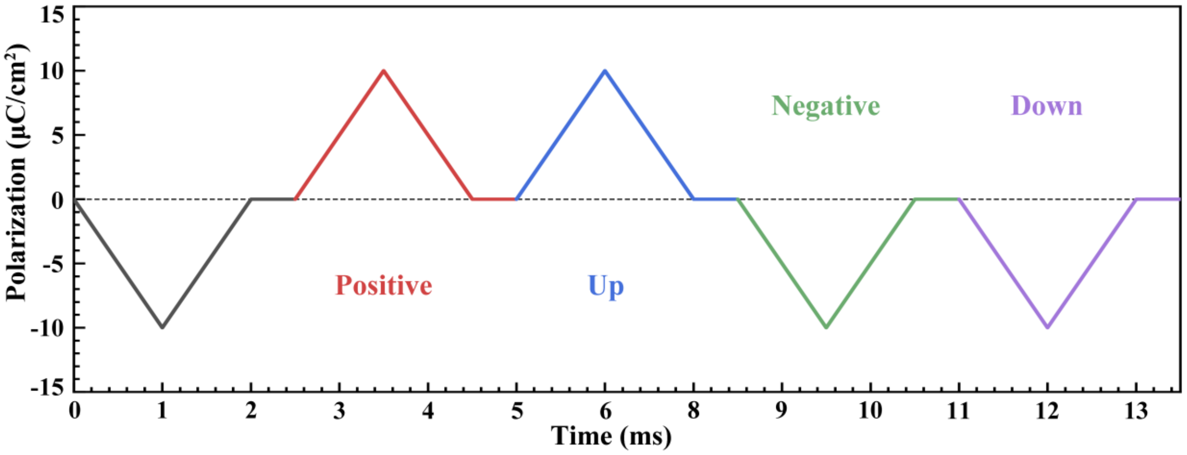

Supplementary Fig. S12 | Parameters of positive-up-negative-down (PUND) measurements of the SATO films. The write and read voltages are both 10V.

Supplementary Figure S13

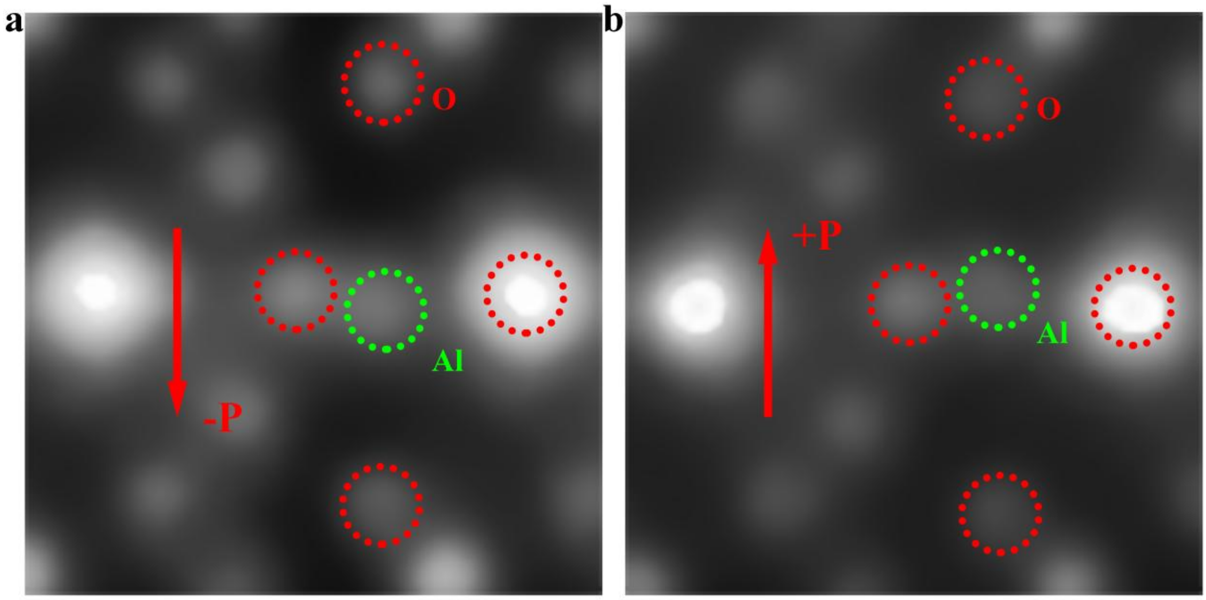

Supplementary Fig. S13 | Atomic origin of the ferroelectricity of SATO. (a) and **(b)** HAADF images showing the upward and downward polarities, respectively. The red and green dotted circles represent the O and Al atoms in the AlO_5 bipyramid, respectively. The upward and downward displacement of Al atoms inside the AlO_5 bipyramids leads to the emergence of ferroelectricity.

Supplementary Figure S14

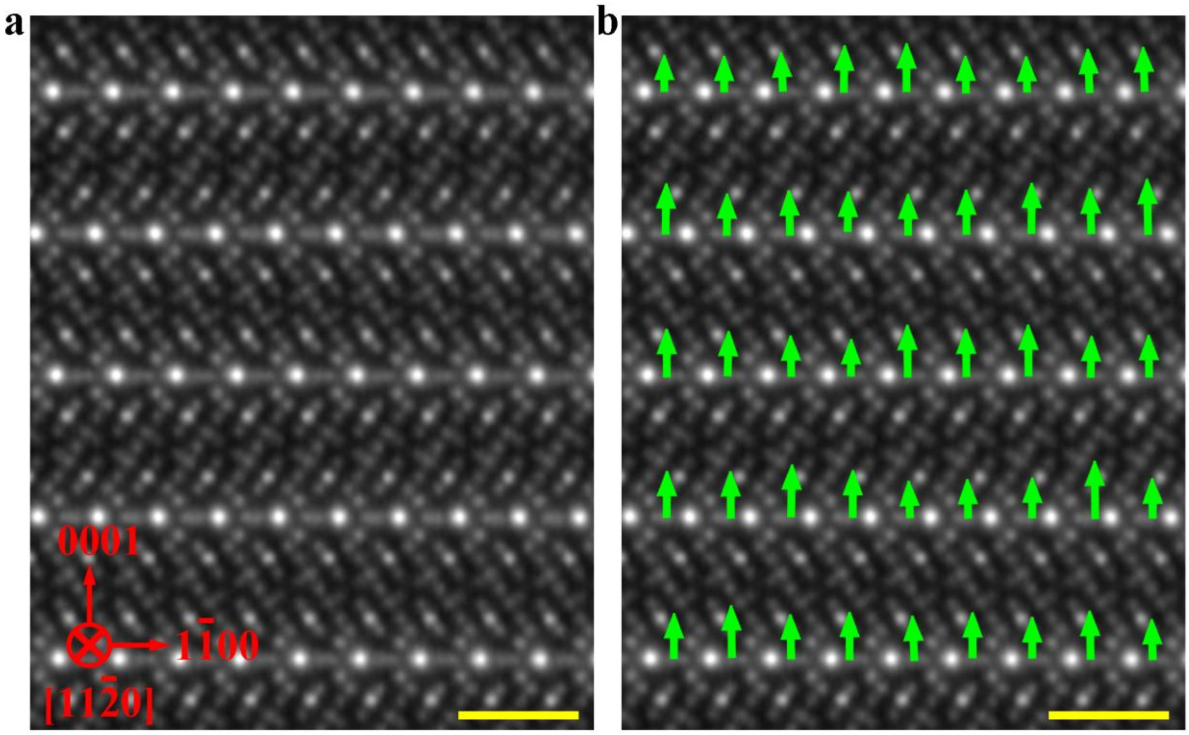

Supplementary Fig. S14 | Analysis of the ferroelectric polarization in SATO. (a) HAADF STEM image of SATO along [11$\bar{2}$0] axis zone. **(b)** Quantitative analysis of polarization displacement in the HAADF image. The direction and length of the green arrows represent the displacement of Al atoms inside the AlO_5 bipyramids. The length of the arrows has been magnified by 25 times to visualize more clearly the displacement of Al atoms. The displacement of Al atoms in this image ranges from 7 to 15 pm. Scale bar, 1nm.

Supplementary Figure S15

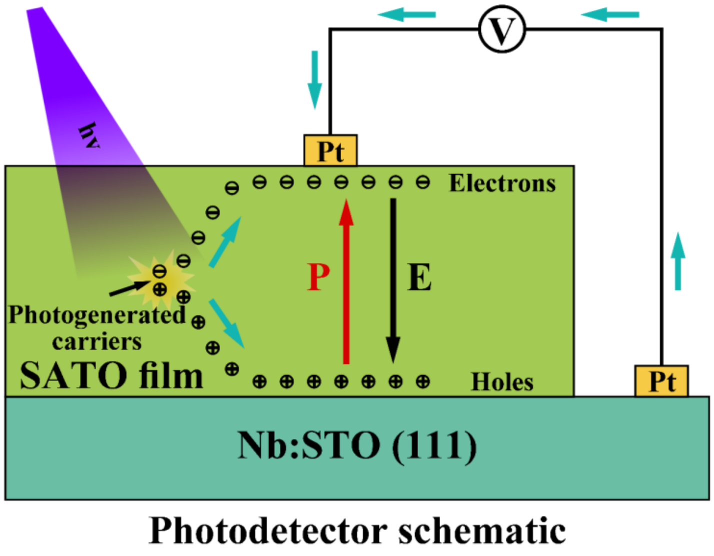

Supplementary Fig. S15 | Schematic diagram of the SATO photodetector test. The SATO thin film generates a spontaneous built-in electric field due to the spontaneous polarization. The photogenerated carriers (electrons and holes) are effectively separated due to the built-in electric field, thereby realizing the conversion of optical signals into electrical signals.

Supplementary Figure S16

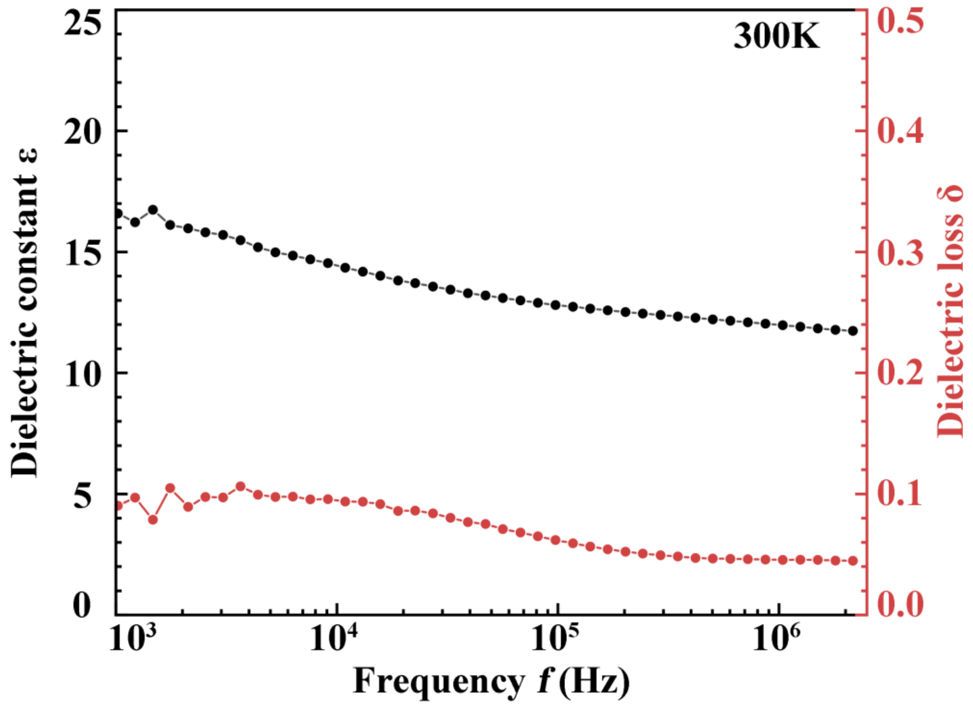

Supplementary Fig. S16 | Dielectric constant and dielectric loss as a function of frequency in the SATO thin film. The SATO film exhibits a large dielectric constant, low dielectric loss, and excellent frequency stability at room temperature.

Supplementary Figure S17

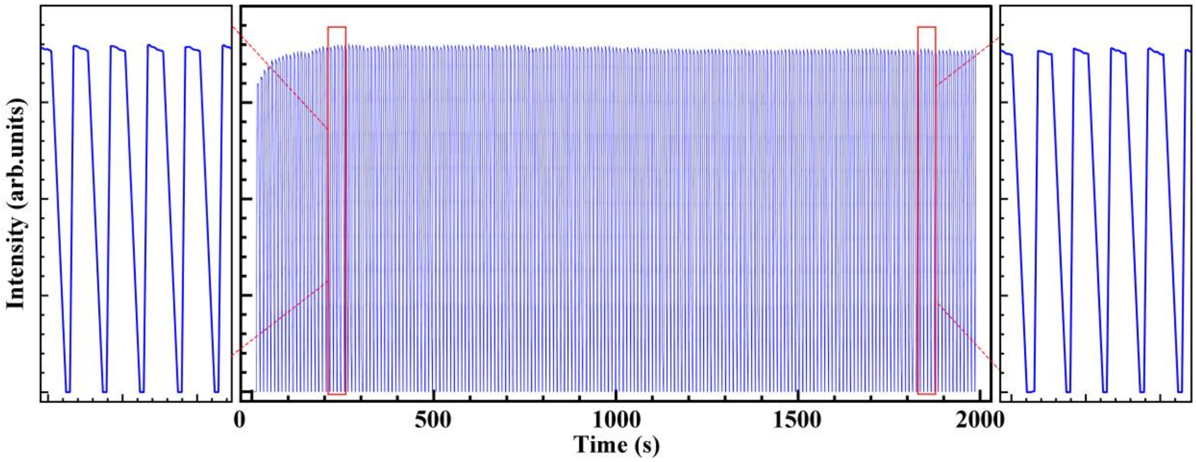

Supplementary Fig. S17 | Long-term stability of the SATO photodetector under 330 nm laser illumination with a power intensity of 50 μW/cm^2. There is almost no significant change in the current, indicating the excellent stability and reliability of the SATO photodetector.

Supplementary Figure S18

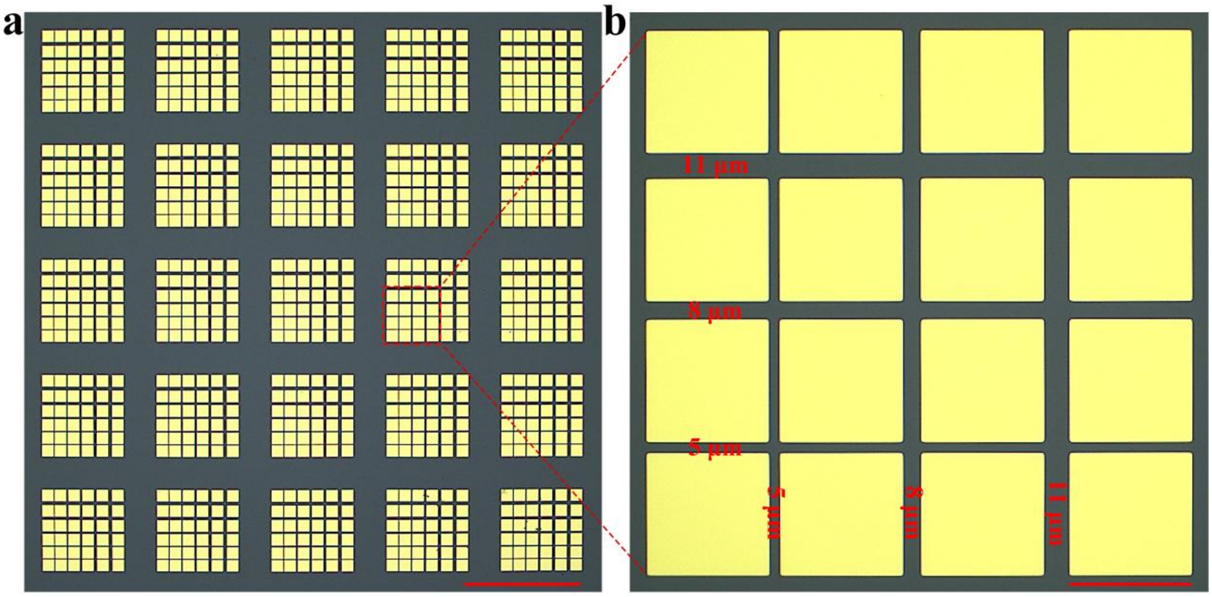

Supplementary Fig. S18 | Optical microscope images of the SATO photodetectors fabricated by photolithography. The size of the Pt electrode is 50 μm × 50 μm. Scale bar, 500 μm in **(a)** and 50 μm in **(b)**.

Supplementary Figure S19

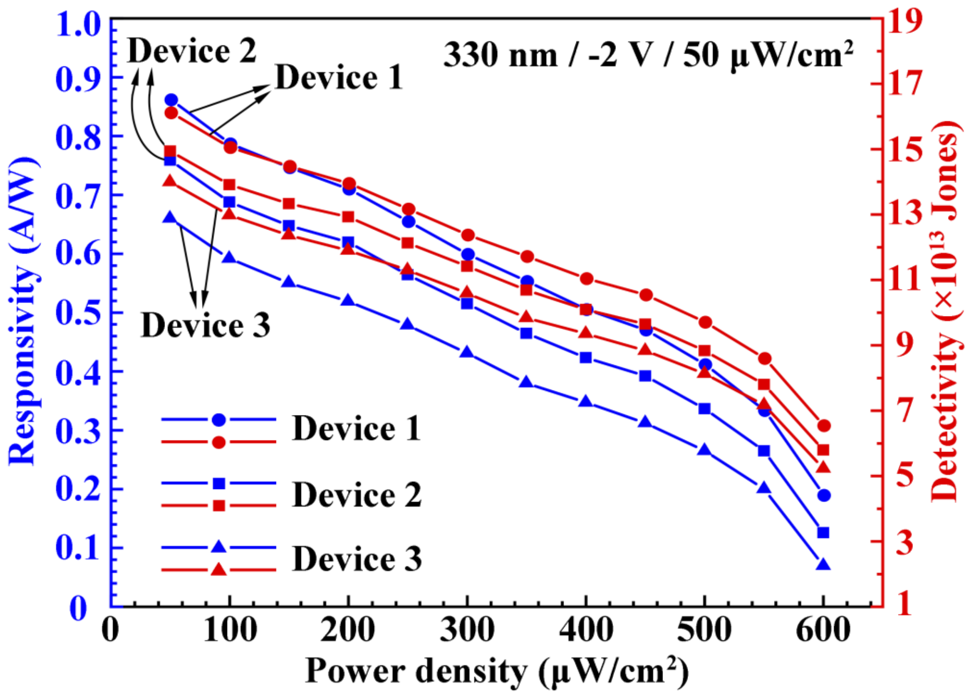

Supplementary Fig. S19 | Responsivity and detectivity curves at different laser power density. All the devices have excellent detection capabilities of weak light.

Supplementary Figure S20

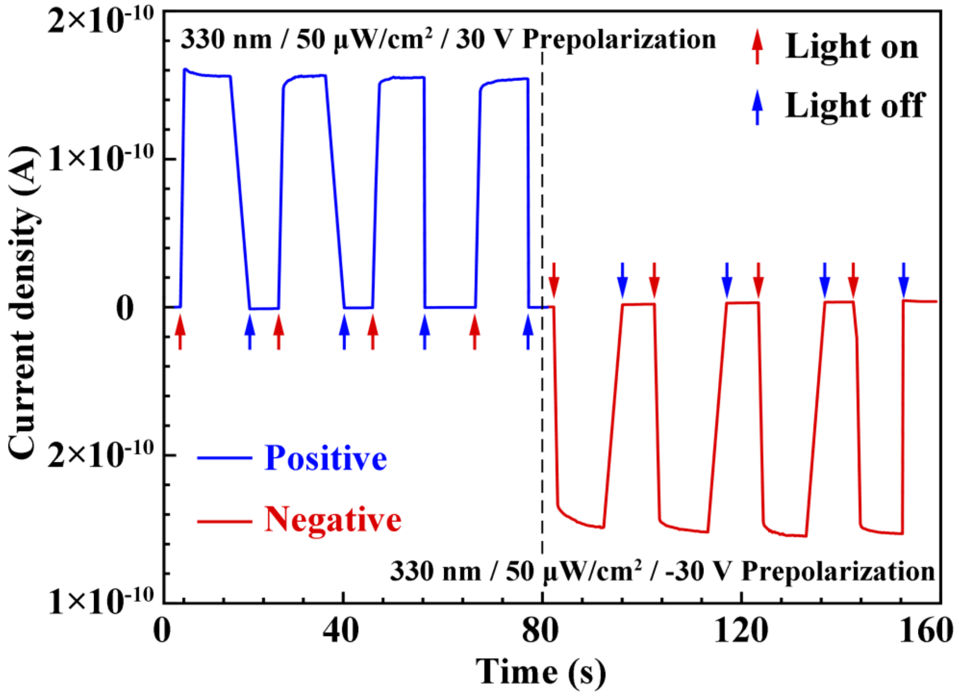

Supplementary Fig. S20 | I-T curves under 330 nm on-off illumination at positive and negative polarizations of the SATO photodetector. The photocurrents under positive and negative polarizations are almost equal.

Supplementary Figure S21

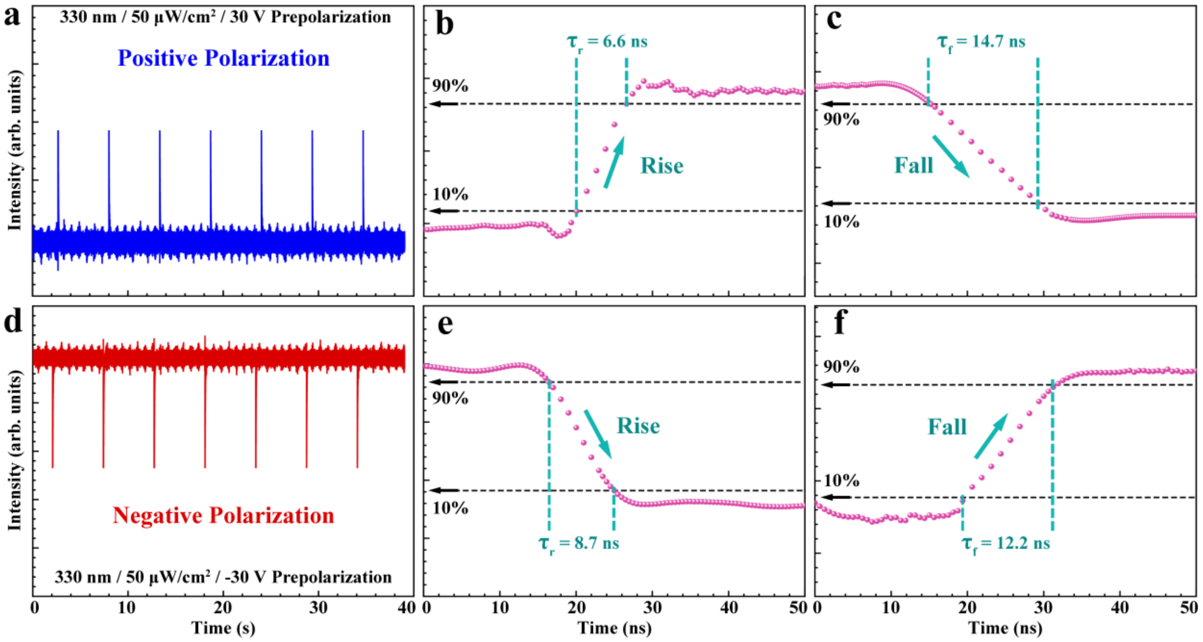

Supplementary Fig. S21 | Time-resolved transient photoresponse curves of the SATO photodetectors with positive and negative polarizations. The SATO photodetectors with positive and negative polarizations have ultra-fast response speed. Their rise/fall times are 6.6 ns/17.7 ns **(a-c)** and 8.7ns/12.2 ns **(d-e)**, respectively.

Supplementary Table S1

Table S1 | Quantitative statistics of EDS spectra in different areas of SATO films.

	Sr (Norm.at.%)	Al (Norm.at.%)	Ti (Norm.at.%)	O (Norm.at.%)	Sr : Al : Ti : O
SATO_EDS_#1	3.15	33.76	3.24	59.85	1.00 : 10.72 : 1.03 : 19.00
SATO_EDS_#2	3.12	33.35	3.24	60.29	1.00 : 10.69 : 1.04 : 19.32
SATO_EDS_#3	3.18	34.09	3.13	59.60	1.00 : 10.72 : 0.98 : 18.74

Supplementary Table S2

Table S2 | Statistical summary of the optoelectronic performance of SATO photodetectors.

	I_d (nA)	I_p (nA)	S (cm^2)	P ($\mu W/cm^2$)	R (mA/W)	D* (Jones)	Response time (ns)
**Device 1 (No polarization)**	2.10×10^{-5}	1.08×10^{-1}	2.5×10^{-6}	50	860.00	1.63×10^{13}	6.8/17.7
**Device (Positive polarization)**	2.25×10^{-5}	1.09×10^{-1}	2.5×10^{-6}	50	871.82	1.62×10^{13}	6.6/14.7
**Device 1 (Negative polarization)**	1.80×10^{-5}	9.08×10^{-2}	2.5×10^{-6}	50	726.26	1.51×10^{13}	8.7/12.2
**Device 2**	4.02×10^{-5}	1.53×10^{-1}	4.0×10^{-6}	50	764.67	1.35×10^{13}	9.9/21.3
**Device 3**	5.15×10^{-5}	1.75×10^{-1}	5.5×10^{-6}	50	636.18	1.16×10^{13}	15.5/30.0

Defects in ferroelectric materials will induce a notable lateral broadening of the hysteresis loop as the applied electric field or frequency increases beyond the saturation threshold, which in turn leads to a gradual increase in coercivity,[1,2,3] as shown in Fig. 4d. The factors contributing to the increase in coercivity of ferroelectric materials are the intrinsic defects (e.g., vacancies) and extrinsic defects (e.g., heterointerfaces), which can act as pinning centers that anchor ferroelectric domain walls, thereby impeding their dynamic switching behavior.[4,5,6] As illustrated in the experimental results, when the applied voltage was increased from 10 V to 12 V, the P-E loop exhibited a significant lateral broadening, while the saturation polarization remained nearly unchanged. This experimental observation is well-aligned with the characteristic behaviors of the defect pinning mechanism.

References

1. Balashova, E. *et al.* Structural properties and dielectric hysteresis of molecular organic ferroelectric grown from different solvents. *Crystals* 11, 1278 (2021).
2. Hao, P. *et al.* Highly enhanced polarization switching speed in HfO_2-based ferroelectric thin films via a composition gradient strategy. *Adv. Funct. Mater.* 33, (2023).
3. Shcherbakov A. *et al.* Evolution of coercive voltage in a ferroelectric memory cell based on $Hf_{0.5}Zr_{0.5}O_2$ during its lifetime. *Phys. Rev. Appl.* **22**, 064083 (2024).
4. Zhang, D. *et al.* Superior polarization retention through engineered domain wall pinning. *Nat. Commun.* 11, 349 (2020).
5. Gao, P. *et al.* Revealing the role of defects in ferroelectric switching with atomic resolution. *Nat. Commun.* **2**, 591 (2011).
6. Bencan, A. *et al.* Domain-wall pinning and defect ordering in $BiFeO_3$ probed on the atomic and nanoscale. *Nat. Commun.* 11, 1762 (2020).
